# Nano zero valent iron (nZVI) particles for the removal of heavy metals (Cd^2+^, Cu^2+^ and Pb^2+^) from aqueous solutions

**DOI:** 10.1039/d1ra01427g

**Published:** 2021-05-24

**Authors:** Mekonnen Maschal Tarekegn, Andualem Mekonnen Hiruy, Ahmed Hussen Dekebo

**Affiliations:** Ethiopian Civil Service University, Department of Environment and Climate Change Addis Ababa Ethiopia Mekonnen.maschal@ecsu.edu.et maschalm12@gmail.com; Addis Ababa University, College of Natural and Computational Sciences, Centre for Environmental Science Addis Ababa Ethiopia

## Abstract

For the past 15 years, nanoscale metallic iron (nZVI) has been investigated as a new tool for the treatment of heavy metal contaminated water. The removal mechanisms depend on the type of heavy metals and their thermodynamic properties. A metal whose redox potential is more negative or close to the reduction potential of Fe(0) is removed by the reduction process, while the others will be mediated by precipitation, complexation or other sorption processes. This review summarises our contemporary knowledge of nZVI aqueous chemistry, synthesis methods, mechanisms and actions (practical experiences) of heavy metal (Cd, Cu and Pb) removal and challenges of nZVI practical applications. Its inner core (iron(0)) has reducing ability towards pollutants, while the iron oxide (FeO) outer shell provides reaction sites for chemisorption and electrostatic interactions with heavy metals. Emerging studies highlighted that nZVI surfaces will have negatively charged species at higher pH and have good affinity for the removal of positively charged species such as heavy metals. Different sizes, shapes and properties of nZVI have been produced using various methods. Ferric salt reduction methods are the most common methods to produce stable and fine graded nZVI. Higher uptake of copper(ii), lead(ii) and cadmium(ii) has also been reported by various scholars. Practical pilot tests have been conducted to remove heavy metals, which gave highly satisfactory results. Challenges such as agglomeration, sedimentation, magnetic susceptibility, sorption to other fine materials in aqueous solution and toxicity of microbiomes have been reported. Emerging studies have highlighted the prospects of industrial level application of nano zero valent particles for the remediation of heavy metals and other pollutants from various industries.

## Abstract 2

Heavy metals (Cd^2+^, Cu^2+^ and Pb^2+^) removal mechanisms of nZVI from aqueous solution.

## Graphical abstract

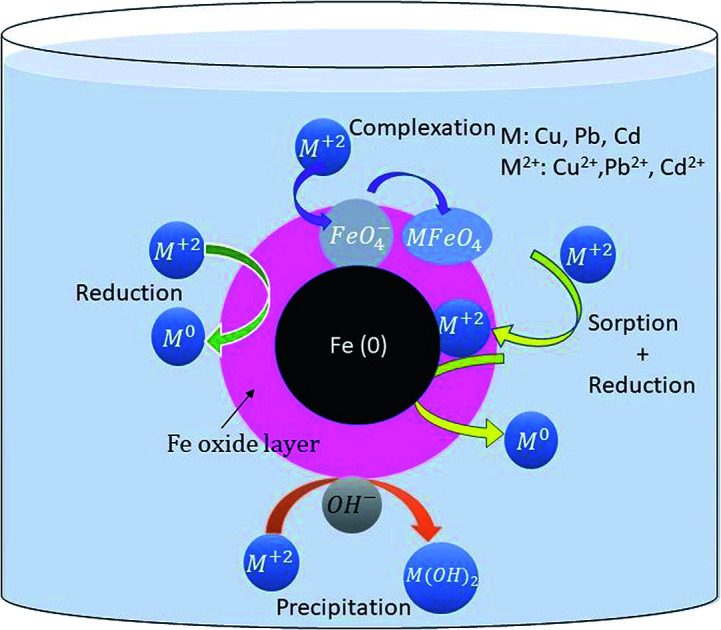

## 1. Introduction

The increasing rate of industrial development has induced the release of heavy metals, such as lead, copper, cadmium and others, and has created an in-evitable environment. Many technologies have been developed to reduce these pollutants before reaching to water bodies. Nanotechnology is among the recently introduced heavy metal removal techniques that has attracted a lot of attention of many scientists.^1–6^ Various nanomaterials such as multi-walled carbon nanotubes, nanoparticles and fabricated composites were utilized to remove heavy metal ions from contaminated industrial wastewater.^6–10^ The unique characteristics such as stability, less toxicity, high specific surface area and high pore size with high heavy metal adsorption capacity make nanomaterials excellent candidates for the clean-up of toxic heavy-metal ions from industrial wastewater.^11–13^

Nano zero valent iron was extensively studied for its ability to remediate heavy metal contaminated wastewater effluents from different industries. It's less toxicity and comparative abundance in nature led nZVI to wide applications in the field of environmental pollution abatement.^1,14,15^ Nano-zero valent iron (nZVI) has Fe(0) core and Fe oxide outer shell, wherein the core has reducing ability and the outer shell acts as a reaction site for chemisorption and electrostatic interactions. Mechanisms such as reduction, absorption, precipitation and mineralization have played a role in the removal of heavy metals from aqueous phases using nZVI.^1,15^ When dissolved in water nZVI exhibits ligand like properties. At low pH, iron oxide shell is positively charged and attracts anions like phosphates and sulphates, and at high pH it becomes negatively charged and attracts cations like metal ions. Heavy metals are sequestered onto the solid nZVI surface after the reaction.^1^ In this paper, different articles that have been published on various methods of nZVI synthesis, a laboratory scale and field level practical tests of heavy metal removal capacity of nZVI were collected and reviewed.

## 2. Chemistry of iron and its natural abundance

### 2.1 Abundance of iron and its chemical characteristics

Iron is relatively abundant in the earth's crust.^16,17^ It has been reported in the literature that iron is the second most abundant metal next to aluminium in igneous rocks. The element is a principal constituent of many igneous rocks, especially those containing basic silicate minerals. Divalent iron links the chains of silicon-oxygen tetrahedra in minerals such as pyroxenes and amphiboles and links individual tetrahedron in the structure of fayalite. Trivalent iron replaces aluminium in some silicate minerals. Also, iron is common in the form of oxide and sulphide.

Sedimentary rocks contain iron in various forms, but ferric oxides are perhaps more common. Geochemical data show that iron weathered out of solid minerals is not retained for long in solution in water but is redeposited as solid oxides or hydroxides. Iron occurs in two oxidation states, the divalent or ferrous form and the trivalent or ferric form.

**Fig. 1:**
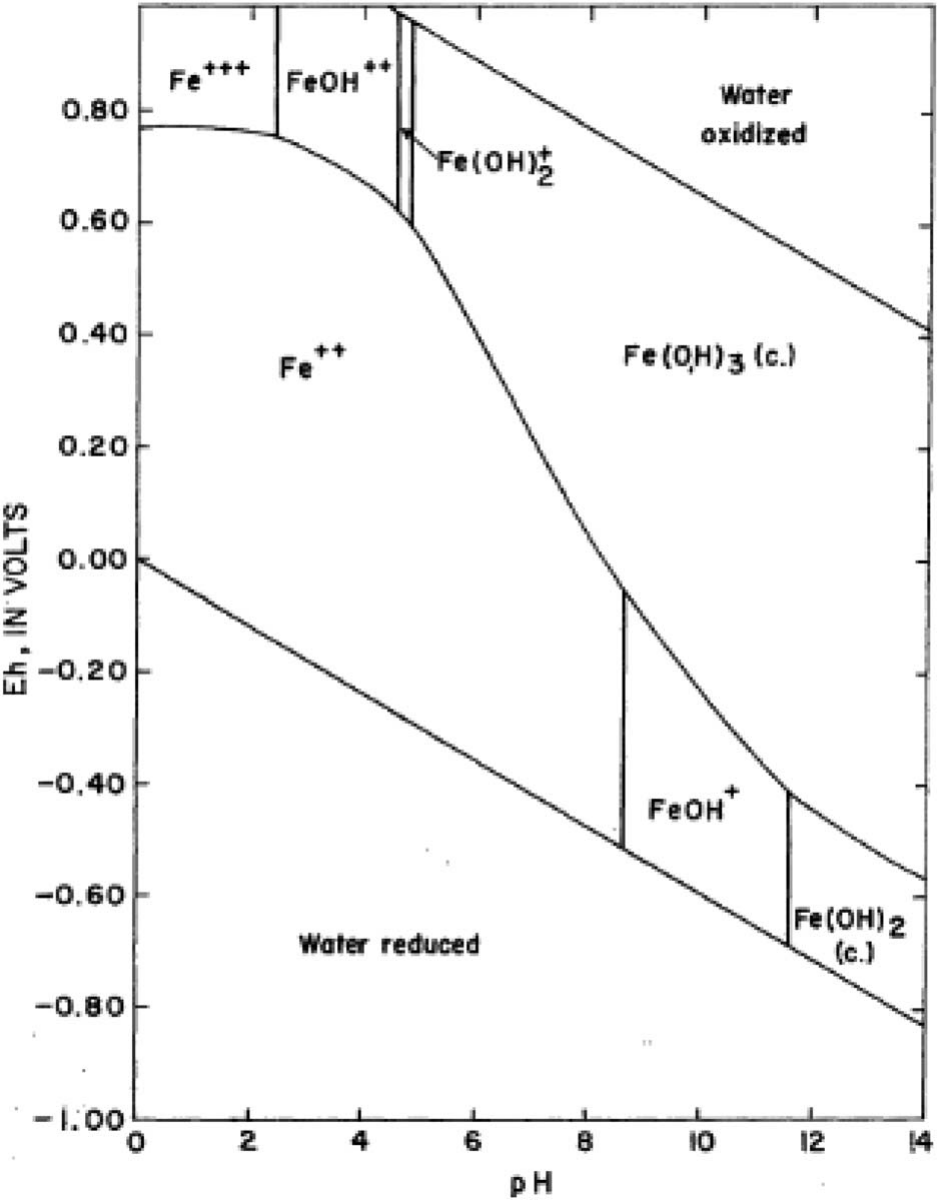
Stability-field diagram for aqueous ferric-ferrous system; adapted from Horr (1962).^18^ The most common species of iron in natural waters is ferric hydroxide, Fe(OH)_3_ (or more correctly, Fe_2_O_3_·3H_2_O). At equilibrium in the pH range of 5 to 8, this compound is largely in the solid state, the solubility being very low. Ferric hydroxide is a weak base and ionizes as Fe(OH)_2_^+^, FeO^+^, FeOH^++^, and Fe^+++^. If the pH is sufficiently high, anions such as ferrite, FeO_2_^−^, are formed. Higher oxidation states are also possible, FeO_4_^−^ being the best known of these species. Ferric iron is a powerful former of complexes.

Iron in aqueous solution is subject to hydrolysis. Iron hydroxides are formed, especially the ferric form with very low solubility. The retention of iron in solution is consequently affected by the pH of the solution. In most natural waters, the pH is not low enough to prevent hydroxides from forming, and under oxidizing conditions, practically all the iron is precipitated as ferric hydroxide. Another important feature of the chemical behaviour of iron in solution is its tendency to form complex ions with inorganic ligands (Fig. 1).

In natural water, ZVI nanoparticles can exhibit metal-like or ligand-like coordination properties depending on the solution chemistry. Iron(0) particles are covered with iron oxides. At lower tract when the pH is lower than its point of zero charge pH (<pHzpc = 8), the iron oxides (nZVI surface cover) are positively charged whereas when the solution pH is above the iso-electric point, the oxide surface becomes negatively charged and can form surface complexes with cations (*e.g.*, metal ions). At higher pH above its iso-electric point, zero-valent iron can serve as very effective electron donors (*i.e.*, reductants) with nZVI (Fe^2+^/Fe) standard reduction potential (*E*^0^) of −0.44 V, which is lower than many metals such as Pb, Cd, Ni, and Cr. As a result, it will then bind heavy metals.

### 2.2 Aqueous chemistry of nZVI (Fe^0^)

Metallic iron (Fe^0^), also referred to as zero-valent iron, is well recognised as being highly susceptible to oxidation in aqueous media. Its oxidation is considered to occur primarily through an electrochemical process, with anodic and cathodic components. The anodic reaction involves the dissolution of Fe (forming soluble ionic products or insoluble oxide/hydroxide) and is coupled with the reduction of redox amenable species at the cathode. In natural waters, the primary components available for oxidation reactions are dissolved oxygen (DO) and water, with the former being thermodynamically favoured (eqn (1) and (2)).

1 2Fe^0^_(s)_ + 4H_(aq)_^+^ + O_2(aq)_ → 2Fe^2+^ + 2H_2_O_(l)_*E*^0^ = +1.67 V

2 2Fe^0^_(s)_ + 2H_2_O_(l)_ → 2Fe^2+^ + H_2(g)_ + 2OH_(aq)_^−^*E*^0^ = −0.39 V

Ferrous ion (Fe^2+^) is a primary product from these reactions and, in turn, can undergo further oxidative transformation (eqn (3) and (4)).

3 2Fe_(s)_^2+^ + 2H_(aq)_^+^ + ½O_2(aq)_ → 2Fe^3+^ + H_2_O_(l)_*E*^0^ = +0.46 V

4 2Fe_(s)_^2+^ + 2H_2_O_(l)_ → 2Fe^3+^ + H_2(g)_+ 2OH_(aq)_^−^*E*^0^ = −1.60 V

As described in the above reactions, an increase in solution pH tends to consume protons or produce hydroxyl ions. The high reactive surface area of nZVI (up to 100 m^2^ g^−1^) rapidly facilitates the chemically reducing conditions when the mass of material is added to an aqueous system (eqn (2) and (4)).^19^

### 2.3 Core–shell model theory

**Fig. 2:**
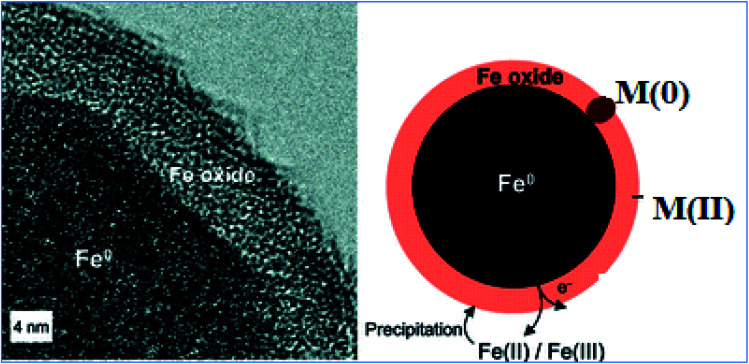
Cross-sectional structure of nZVI; adapted from Ramos *et al.* (2009).^23^

In actual conditions, nZVI is covered with an oxide layer (Fig. 2). According to the core–shell model theory, the iron oxide shell is largely insoluble under neutral pH conditions and may protect the nZVI core from rapid oxidation.^20^ The composition of the oxide shell may depend on the fabrication processes and environmental conditions. For example, the oxide shell of α-Fe nanoparticles generated by sputtering consists mainly of maghaemite (γ-Fe_2_O_3_) or partially oxidized magnetite (Fe_3_O_4_). Nanoparticles formed *via* nucleation of metallic vapor also contain γ-Fe_2_O_3_ and Fe_3_O_4_, with more γ-Fe_2_O_3_ for smaller particles due to the higher surface-to-volume ratio and rapid surface oxidation. On the other hand, particles produced by hydrogen reduction of goethite and hematite particles reportedly have only Fe_3_O_4_ in the shell.^21,22^ The presence of wustite (FeO) has also been noted. It is not clear from the existing literature whether variations in the shell structure and composition have any effect on the iron nanoparticle reactivity, aggregation, and transport.

## 3. Methods of nZVI synthesis

There exist several physical and chemical methods for the synthesis of nZVI such as grinding, abrasion, lithography, nucleation from homogeneous solutions or gas, annealing at elevated temperatures and reacting with reducing agents. These synthesis methods are grouped under two broad approaches: the bottom-up and top-down approaches. The bottom-up approach involves assembling individual atoms and molecules to form nano-sized structures; it uses a wide range of reductants to convert dissolved iron in solution to nZVI. Whereas, the top-down approach on the contrary involves the crushing of bulk particles (microscale or granular) of iron to fine nano-sized particles by mechanical or chemical ways.^24^ The choice of synthesis method influences both the size and shape of the nano particles produced.

### 3.1 Liquid-phase reduction

**Fig. 3:**
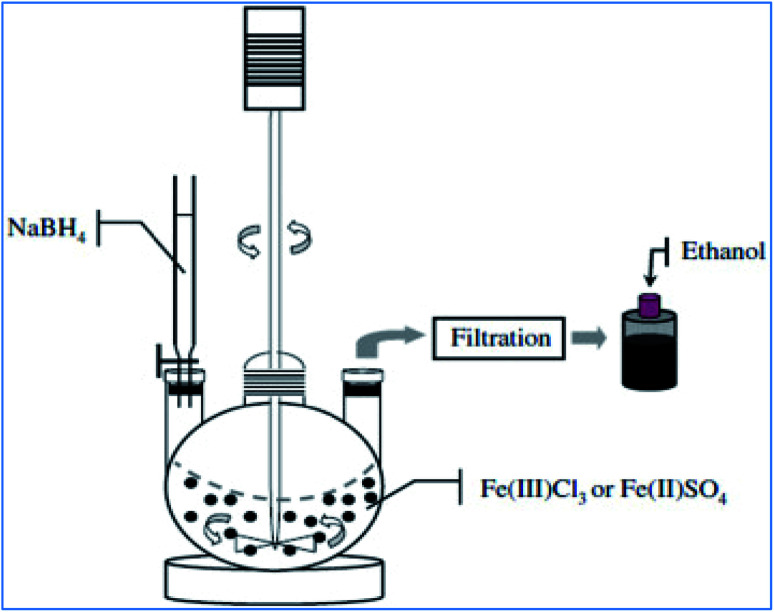
Liquid phase metal reduction method for nZVI preparation; adapted from Jamei *et al.* (2012).^26^

The synthesis of nZVI can be achieved by reacting iron salts or compounds with a strong reducing agent such as sodium borohydride (NaBH_4_). The borohydride solution prepared from concentrated ethanol and NaBH_4(s)_ is slowly added into the iron (ferric or ferrous) salt solution while stirring vigorously under anaerobic conditions (Fig. 3). The resulting black precipitate is vacuum filtered, washed with deionized water or ethanol and subsequently dried.^25^ As shown in eqn (5) and (6), iron(iii) reduced to iron(ii); then it converts to nZVI in the presence of excess reducing agents.

5 4Fe_(aq)_^3+^ + BH_4_^−^ + 3H_2_O → 4Fe_(s)_^2+^ + H_2_BO_3_^−^ + 4H_(aq)_^+^ + 2H_2(g)_

6 2Fe_(aq)_^2+^ + BH_4_^−^ + 3H_2_O → 2Fe^0^_(s)_ + H_2_BO_3_^−^ + 4H_(aq)_^+^ + 2H_2(g)_

7 4Fe_(aq)_^3+^ + 3BH_4_^−^ + 9H_2_O → 4Fe^0^_(s)_ + 3H_2_BO_3_^−^ + 12H_(aq)_^+^ + 6H_2(g)_

### 3.2 Gas-phase reduction methods

This process uses gases such as H_2_, CO_2_ or CO acting as reducing agents to reduce iron salts or compounds to nano form at high temperature (>500 °C). The particles that are produced in these methods have an average particle size ranging from 50 to 300 nm with a specific area of about 7.55 m^2^ g^−1^.^27^ The reaction proceeds according to eqn (8) and (9). The method is friendly to humans and the environment as it does not generate any secondary toxic pollutants. However, it is energy intensive and demands unique equipment to get nano-sized iron(0) particles. Also, the method leads to a speciation of iron and produce various form of iron nanoparticles at different proportions.

8 Fe(C_2_H_3_O_2_)_2(aq)_ + C_(g)_ → Fe^0^_(s)_ + 2CH_2_CO + CO + H_2_O

9 Fe_3_O_4(aq)_ + 2C_(g)_ → 3Fe^0^_(s)_ + 2CO_2_

### 3.3 Thermal decomposition methods for nZVI synthesis

One synthesis method which is well-known for producing high quality particles with more narrow size distribution is the thermal decomposition of an iron precursor. This method is quite similar to the gas phase reduction previously explained. The iron precursor (iron oxide or an iron salt) is reduced at high temperatures (>500 °C) in the presence of gaseous reducing agents. The reducing agents used for this synthesis include N_2_, H_2_, CO, and Ar.^28,29^

### 3.4 Ultrasound assisted method for the synthesis of nZVI

**Fig. 4:**
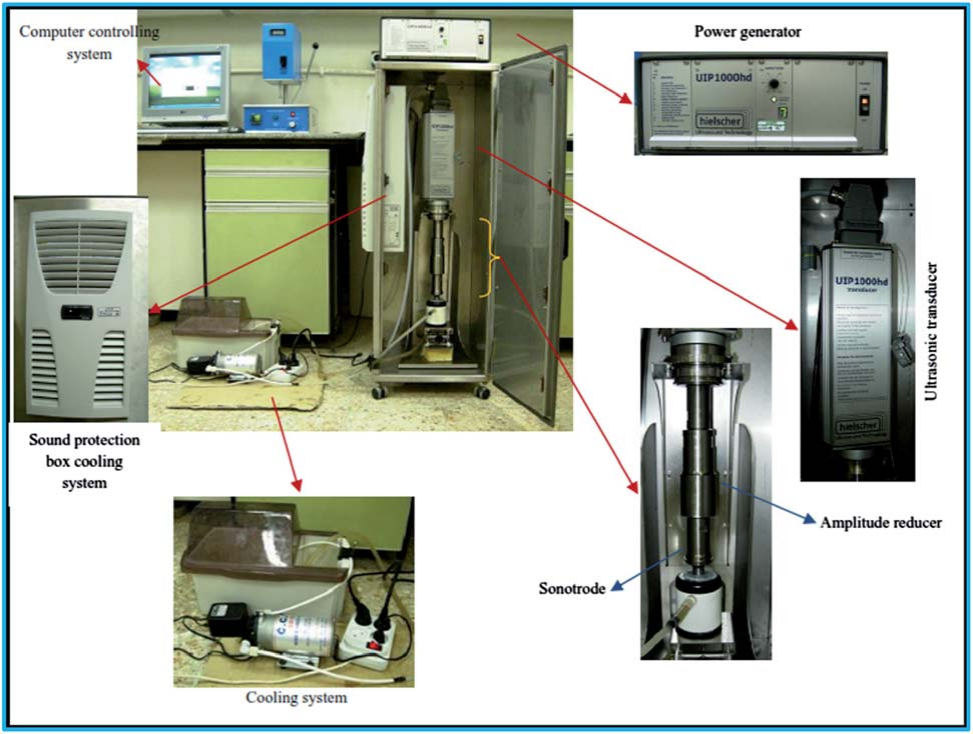
nZVI synthesis using ultrasound sonication process; adapted from Jamei *et al.* (2012).^26^

As it was cited in Stefaniuk *et al.* (2016)_,_^29^ the ultrasound assisted nZVI syntehsis method was first developed and tested by Tao *et al.* in the State Key Laboratory for RSA, Institute of Metal Research, Chinese Academy of Sciences in 1999. The method helps to enhance the physical and chemical properties of nZVI (eqn (10)). This method uses reducing agents like sodium borohydride and ammonium hydroxide to produce small, uniform and equal-axe grains of iron nanoparticle of average size of 10 nm.^30^ As show in Fig. 4, the ultrasound sonication process increased the temperature of the system and reduced the reaction time to get nZVI particles. The study observed decreasing particle size with increasing precursor concentration and ultrasonic power. In addition, the surface area of nZVI was increased when applying ultrasonic vibration.

10 4Fe_(aq)_^2+^ + BH_4(aq)_^−^ + 7NH_4_OH → 4Fe^0^ +H_3_BO_3(aq)_ + 7NH_4(aq)_^+^ + 4H_2_O

### 3.5 Precision milling method for nZVI synthesis

**Fig. 5:**
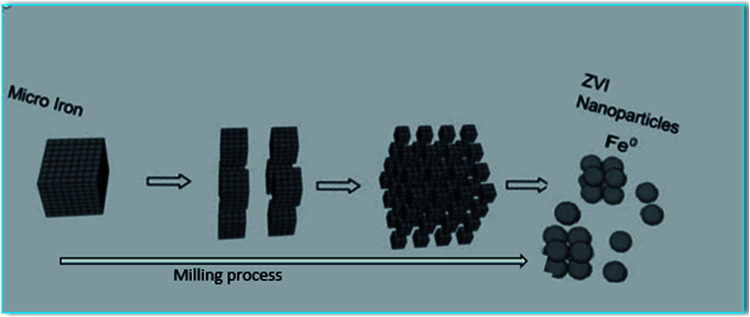
Top-down approaches for nZVI preparation (precision milling).

Precision milling method consists of applying mechanical force to crush micro iron with steel shot in a high-speed rotary chamber for about 8 h without any chemical to achieve highly reactive nanoparticles of diameter 10–50 nm and surface area 39 m^2^ g^−1^ (Fig. 5). Upon contact with the steel shot, the particles are deformed and cracked producing nanoparticles with irregular shapes and with strong tendency to aggregate because of their high surface energy.^23^

### 3.6 Electrochemical method for nZVI synthesis

**Fig. 6:**
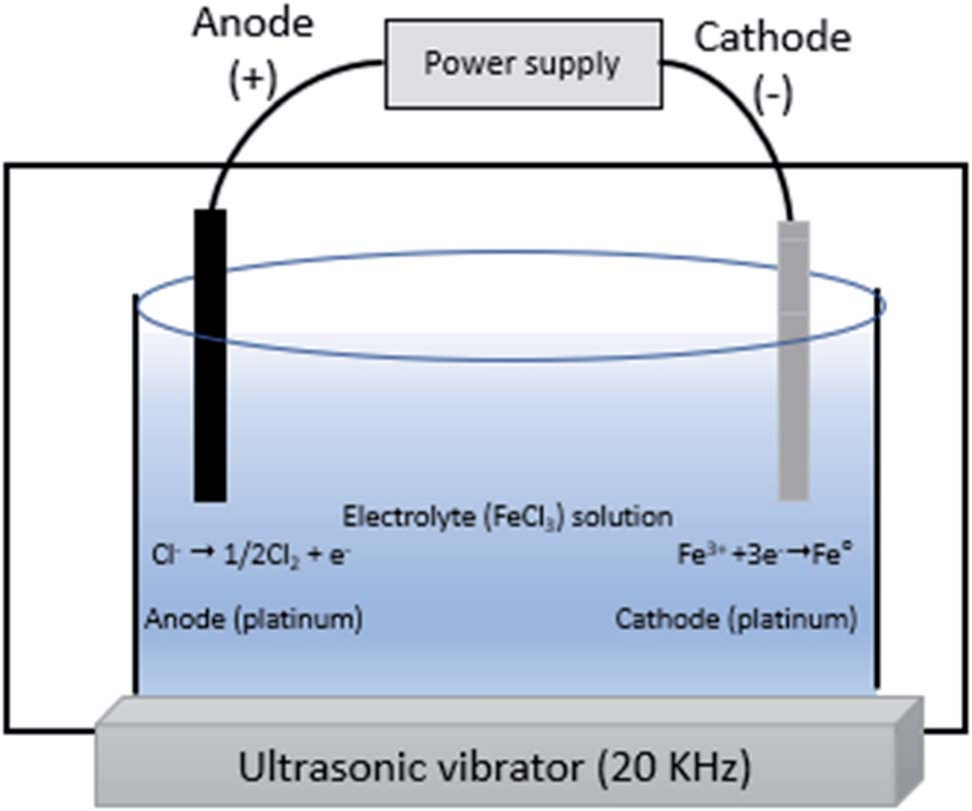
Electrochemical method for nZVI synthesis.

This is a cheap and fast method in which cathodes are used to attract Fe^2+^/Fe^3+^ ions from solution with the help of electric current (Fig. 6). Cationic surfactants are used to act as a stabilizing agent and ultrasonic waves (20 kHz) as a source of energy are necessary for fast removal of iron nanoparticles from the cathode.^29^ This method produces particles of diameter 1–20 nm and specific surface area of 25.4 m^2^ g^−1^.

11 Cathode reaction: Fe^3+^ + 3e^−^ + stabilizer → *n*Fe^0^

### 3.7 Green method for nZVI synthesis

**Fig. 7:**
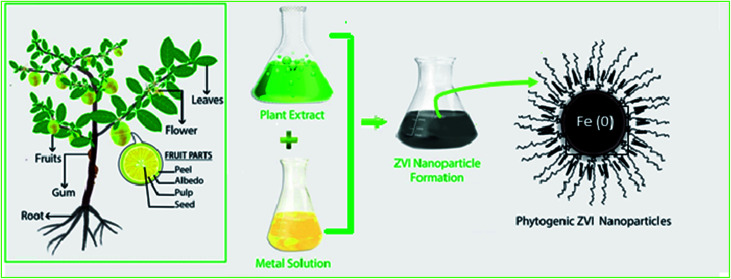
Synthesis of nZVI from plant extracts; modified from Puthukkara *et al.* (2020).^33^

This is an inexpensive and environmentally friendly procedure where highly water soluble, less toxic, and biodegradable plant extracts are used to reduce iron to nanoscale (Fig. 7). The extract is heated in water close to the boiling point and then mixed with the iron ion solution causing the iron ions to be reduced to nZVI in the presence of polyphenols. The extracts serve as a reducing agent and then as a capping agent for Fe. This method produces spherical shape nZVI with the size ranging from 5 to 15 nm.^31,32^

## 4. Adsorption mechanisms of heavy metals on nZVI

**Fig. 8:**
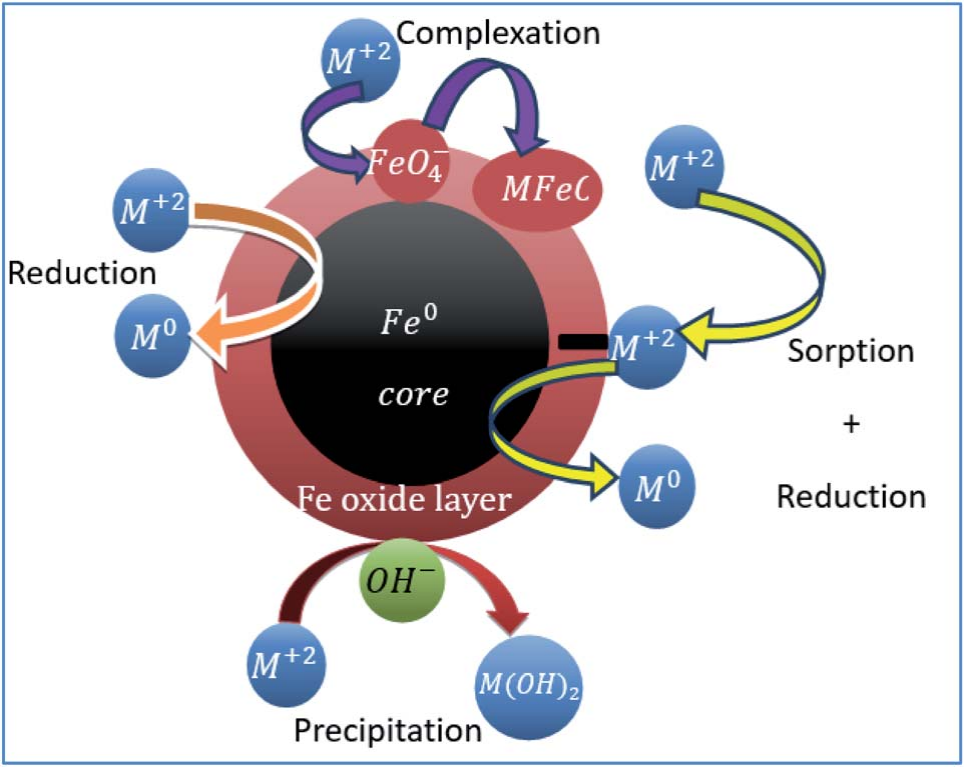
State of nZVI and its cationic removal mechanism in aqueous media.

The availability of active sites and/or many functional groups on nZVI surface and sub surface has been confirmed.^34,35^ The nanoparticles are composed of the core (metallic iron) and the shell, *i.e.*, the oxide layer of Fe(ii) and Fe(iii), which is formed due to the rapid metallic iron oxidation in the environment (Fig. 8). Metallic iron has a reductive power and promotes the reduction of various pollutants in the environment. In aqueous solutions, zero valent iron nanoparticles react with water and oxygen to form an outer layer of iron (hydr)oxide.^20^ The shell has a thickness of at least 3 nm^19,35^ and provides sites for the formation of chemical complexes.^36^ The core (Fe^0^) acts as an electron donor with the following anodic reaction: Fe → Fe^2+^ + 2e^−^.^1,37^ The outer layer of iron (hydro)oxide acts as an effective adsorbent of pollutants, including heavy metals.^20,38^ In water, Fe(0) nanoparticles can show the coordination properties of a metal or ligand depending on the pH of the solution. At pH > 8, the surface of nanoparticles becomes negatively charged and can form surface complexes with heavy metal cations.

The reduction of heavy metals involves two distinct mechanisms; first, the heavy metal is reduced when it comes in contact with Fe(0) in an aqueous solution. Then, the heavy metal is adsorbed onto the core–shell structure of nZVI followed by its gradual reduction by Fe(0).^39^ Heavy metals such as nickel (Ni^2+^) and lead (Pb^2+^) were removed from aqueous solutions by reducing to Ni^0^ and Pb^0^ and adsorbing these cations on iron (hydr)oxide shell.^1^ Also, Pb^2+^ was precipitated to Pb(OH)_2_ and separated from the solution in the form of precipitant. But Pb(OH)_2_ was changed to hydroxide complex at higher pH and setting of the appropriate optimal pH for the lead hydroxide precipitant formaiton was required.^40^ On the other hand, copper adsorption study on nZVI showed the removal of Cu^2+^ from solution by chemical reduction to an elemental form and further sorption on the oxidized surface of nZVI.^41,42^ In general, other competing heavy metal ions affect the reduction process and the co-existing ions such as phosphates, bicarbonates/carbonates, sulphates and nitrates found in solution affect (co)precipitation.^43,44^

The main adsorption mechanisms of common heavy metals found in the surface, ground and wastewater depend on their aquatic chemistry. However, the removal mechanism is mainly dominated by adsorption and other processes such as reduction, hydroxide precipitation and electron transfer. Studies showed that the heavy metals in the solution might undergo one or more of the processes such as surface complexation, physisorption, precipitation and/or reduction.^45,46^

The heavy metals possessing lesser standard redox potential than nZVI (*E*^0^ = −0.44 V) undergo reduction; nZVI serves as an effective electron donor for the metallic ions to be reduced.^47^ The metals whose redox potential (*E*^0^) is more negative or close to that of Fe^0^ (such as Cd) are removed by sorption and/or precipitation.^20^

## 5. Heavy metal (Pb, Cd and Cu) removal efficiency of nZVI

### 5.1 Lead(ii) ion removal efficiency

ZVI nanoparticles have been tested for removal of various heavy metals. It was reported that copper, cadmium and lead were efficiently removed by nZVI adsorbents.^48^ As it was indicated in the same report, the uptake (removal capacity) of Cd, Cu and Pb ions was 4.33–5.56, 5.40–6.94 and 5.41–6.95 mg g^−1^, respectively. The number of adsorbents (nZVI) to be added affects the rate of heavy metal removal. In another study reported by Bagbi *et al.*, almost 100% lead metal ion removal has been recorded using nZVI at following experimental conditions: lead concentration: 50 mg L^−1^; equilibrium time: 15 min; pH 5–6; temperature: 25 °C and NZVI dose: 0.4 g L^−1^.^49^

In this study, it was justified that increasing the pH promotes the rate of adsorption and lead removal. Lead metal ion removal experiment from wastewater has indicated that the pH of the solution where lead ion is found affects the state or species of nZVI adsorbent used for adsorption; at lower pH, the surface oxide layer is positively charged where it is dominated by protonated ions and attract only the positive ions, whereas at higher pH its surface becomes negatively charged and forms surface complexes with heavy metals such as Cd, Cu, Pb and others.^50^ In the report, it was justified that increasing contact time, pH and nZVI dose increases the removal of the contaminant. However, adsorbate concertation has an inverse relation with the removal capacity. Ahmed *et al.* (2017)^50^ have reported about 98% of lead removal from water after 10 hours of contact time. The authors reported that the physical properties, magnetic susceptibility, and conductance of nZVI were correlated with adsorption efficiency.

### 5.2 Copper(ii) ion removal efficiency

Literatures suggest that the uptake mechanism of metal ions by zero-valent iron is dependent mainly on the standard reduction potential and chemical speciation of the adsorbate ion under the operating pH. Earlier studies have indicated that ions with standard reduction potential larger enough than that of Fe are fixed on nZVI *via* an oxidation–reduction reaction in which Fe^2+^ behaves as a reducing agent. The standard reduction potential of Cu (+0.34 V, 298 K) is well above that of Fe (−0.44 V, 298 K), and consequently, the uptake of Cu^2+^ ions would be expected to primarily take place *via* a redox mechanism. The study result reported by Li and Zhang confirmed that Cu^2+^ ions are reduced to Cu^0^ upon exposure to nZVI. The authors haves cross confirmed the actions by analysing the distribution of Cu on the surface of iron nanoparticles (eqn (12) and (13)). As shown in the equations, the complete Cu reduction had not occurred and that some small portion could have remained in Cu^2+^ state. On the other hand, the availability of FeOOH groups at the solution–iron interface suggested the contribution of surface complexation to the removal of Cu^2+^ ion under investigation.

12 Fe^0^ + Cu^2+^ → Fe^2+^ + Cu^0^

13 Fe^0^+ 2Cu^2+^ + H_2_O → Fe^2+^ + Cu_2_O + 2H^+^

Li *et al.* reported that the average Cu(ii) removal efficiency of nZVI at operating conditions of 10.20 g L^−1^ nZVI and an agitation time of 100 min was greater than 96%, and achieved an uptake capacity of 250 mg g^−1^.^45^ Li *et al.* (2017)^67^ reported a 99% copper removal efficiency from aqueous solution in the presence of other heavy metals. This removal efficiency is very high as compared to the removal capacity of Cu(ii) using other adsorbents such as ZVI/sand mixture, Fe_2_O_3_ nanoparticles, grafted silica, wheat bran and spirogyra species (Table 1). Nevertheless, the application of nZVI presents some limitations such as rapid oxidation, rapid aqueous aggregation, production costs and recovery of the nanomaterials with associated contaminants.^19^

**Table 1:** Copper metal removal capacity of various adsorbents

Sorbent	Uptake capacity (mg g^−1^)	Reference (source)
nZVI	250	45
ZVI/sand mixture	13.3	35
Grafted silica	16.5	52
Wheat bran	15	53
Spirogyra species	133.3	54

**Fig. 9:**
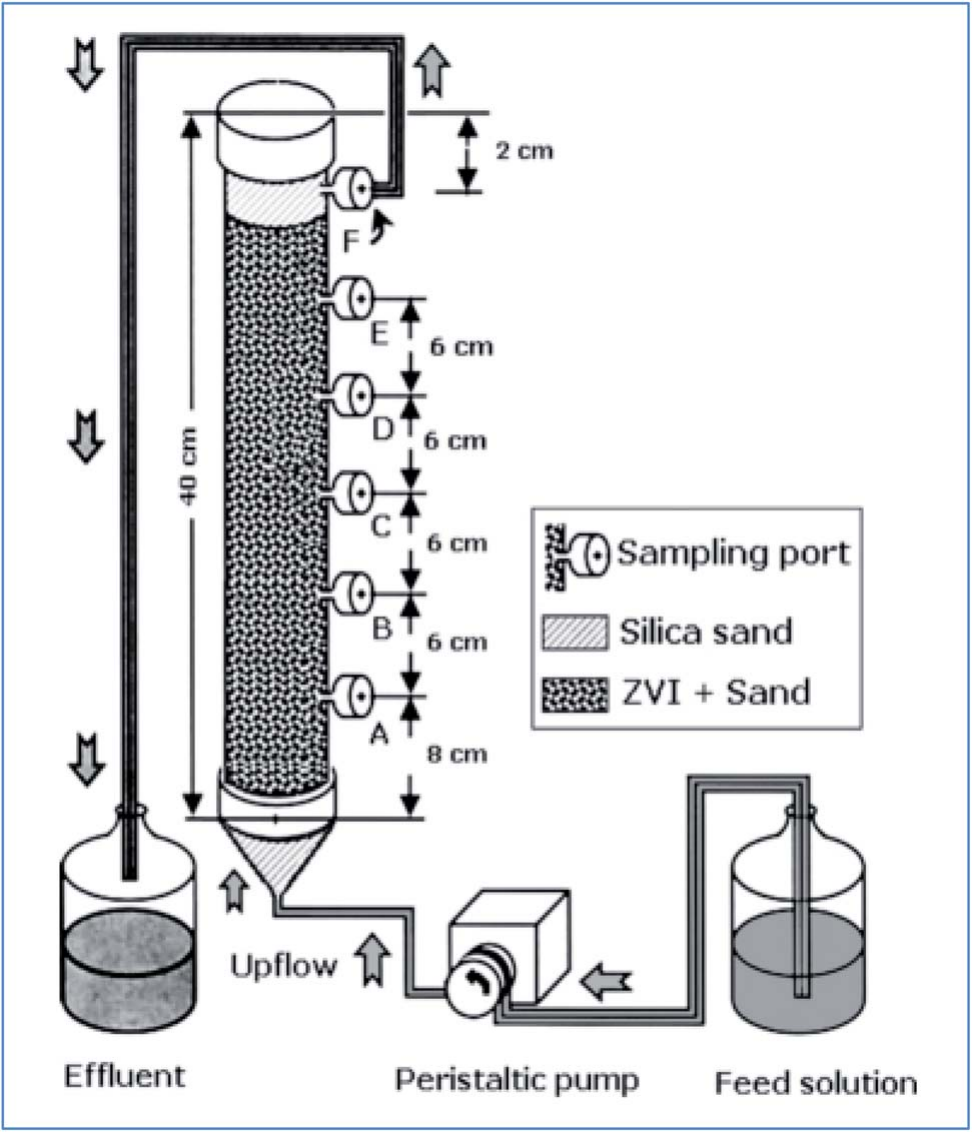
nZVI packed bed column reactor for Cu(ii) treatment; adapted from Komnitsas *et al.* (2007).^51^

Komnitsas *et al.* have tested the copper removal efficiency of nZVI packed bed column reactor (Fig. 9). Accordingly, they have used about 483 g of 0.05 m^2^ g^−1^ ZVI particles to treat 550 mg L^−1^ of Cu(ii) ion under HRT of 6.8 to 34 h, and a volumetric loading rate of 10 g Cu per m^3^ per day. The author has achieved a removal capacity of 0.029 g Cu per g Fe.^51^

### 5.3 Cadmium(ii) removal efficiency

Study has reported Cd metal ion removal capacity of nano zero valent iron (nZVI) by comparing with the removal capacity of other adsorbents that were investigated by other authors (Table 2). According to the study, higher adsorption capacity was found making nZVI the most effective adsorbent for removing Cd from aqueous solutions in comparison to other variety of adsorbents (Table 2). The author stated that the extremely small particle size, large surface area, and high density of reactive and adsorptive sites make nZVI a potential candidate for environmental applications especially for heavy metal (Cd^2+^) removal.^38^

**Table 2:** Cadmium removal capacity of various adsorbents

Type of adsorbent	Adsorption capacity (mmol g^−1^)	Reference
Chitin	0.144	56
Granular activated carbon	0.090	57
Dried activated sludge	1.815	58
Rice husk	0.189	59
Modified kaolinite clay	0.392	60
Nanostructured goethite	0.259	61
nZVI	6.84	38

In 2018, Mahboub Saffari reported a comparative study of cadmium ion adsorption of nZVI to walnut shell biochar modified nZVI; the maximum predicted Cd removal efficiency of the nZVI supported biochar was about 99.72% at initial Cd metal ion concentration of 70.78 mg L^−1^, pH of 6.92, adsorbent dose of 0.56 g L^−1^ and contact time of 40.42 min. The nZVI formed on the biochar *via* adsorption and complexation processes increased the ability of Cd removal than the newly produced biochar–nZVI composite adsorbent.^55^ It was also showed that the equilibrium cadmium adsorption capacity of zero valent iron nano particles with 0.5 g L^−1^ reaches up to 213 mg g^−1^ at 285 K and 225 mg g^−1^ at 333 K.^35^

### 5.4 nZVI modification and adsorption performance enhancement

The adsorption performance of nZVI has been improved by increasing the anionic surfactants for cationic pollutant removal,^41^ increasing surface area to volume ratios,^62^ and reducing the size of particles.^63^ Various researches have been conducted; most of the studies relied on the formation of nZVI supported composites.

The addition of partially acid activated sepiolite on nZVI improved the dispersibility of the adsorbent materials and also formed a porous oxide that can further enhance the heavy metal entrapping action.^64^ nZVI has small particle size and high surface energy, which can adsorb heavy metals or cationic pollutants easily, but it is very difficult to separate it from either sludge or effluent.^65^ Coating Pd on nZVI surfaces has improved the interstitial characteristics of nZVI;^66^ the co-existing organic and inorganic substances impact each of the modified/immobilized nZVI–composites *via* various mechanisms and reduce the targetted heavy metals removal action from the solution. Hence, modifying the nZVI *via* additional support material to improve the effect of co-existing interfering substances is helpful.

## 6. Pilot scale nZVI application for heavy metal removal

### 6.1 Chemical reduction method for Cu(ii) removal from PCB factory wastewater

**Fig. 10:**
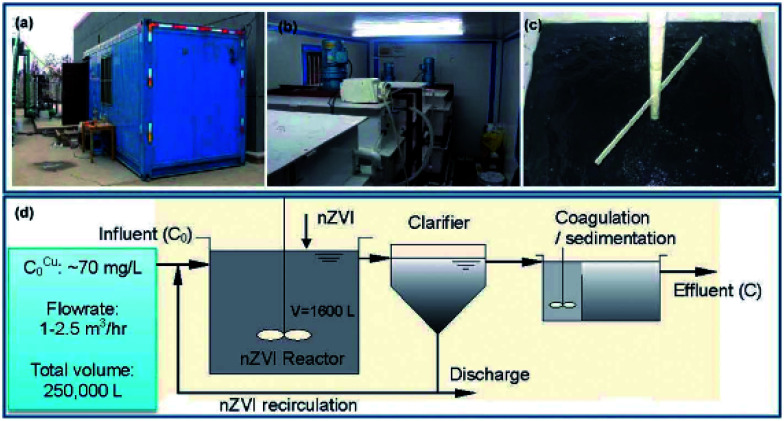
Pilot scale Cu(ii) removal test of nZVI. (a) Container in which the pilot reactors are placed; (b) the nZVI reactor; (c) the nZVI suspension in the reactor; (d) a process flow chart of the pilot test; adapted from Li *et al.* (2014).^45^

Li *et al.* (2014)^45^ conducted a pilot test for the removal of copper using nZVI (Fig. 10). In their research, the influent for the pilot experiment was pumped from the equalization basin of a wastewater treatment facility in a printed circuit board (PCB) factory. The pilot plant and treatment process are shown in Fig. 10. The pilot plant consisted of three tanks, namely a nZVI reactor, a clarifier, and a sedimentation tank, plus additional accessories such as a feeding pump and a sludge recirculation pump. The pH and ORP of the nZVI suspension in the reactor were monitored by online pH and ORP electrodes. The ORP values were corrected with respect to an Ag/AgCl reference electrode and converted to values against the Eh. The pilot tests were conducted for 250 000 L of wastewater containing 70 mg L^−1^ Cu(ii) with a total of 55 kg of nZVI. A completely mixed reactor of 1600 L was operated continuously with flow rates ranging from 1000 to 2500 L h^−1^. The average Cu(ii) removal efficiency was greater than 96% with 0.20 g L^−1^ nZVI and a hydraulic retention time of 100 min.^45^

The nZVI reactor achieved a remarkably high volumetric loading rate of 1876 g Cu per m^3^ per day for Cu(ii) removal, surpassing the loading rates of conventional technologies by more than one order of magnitude. The average removal capacity of nZVI for Cu(ii) was 0.343 g Cu per gram of Fe. The Cu(ii) removal efficiency can be reliably regulated by the solution Eh, which in turn is a function of nZVI input and hydraulic retention time. The ease of separation and recycling of nZVI contribute to process up-scalability and cost effectiveness. As described in eqn (14) and (15), Cu(ii) was reduced to metallic copper and cuprite (Cu_2_O).

14 Cu^2+^ + Fe^0^ → Cu^0^ + Fe^2+^

15 Fe^0^ + 2Cu^2+^ + H_2_O → Fe^2+^ + Cu_2_O + 2H^+^

### 6.2 Chemical co-precipitation, reduction and adsorption methods for Pb(ii) removal from a smelting plant wastewater

**Fig. 11:**
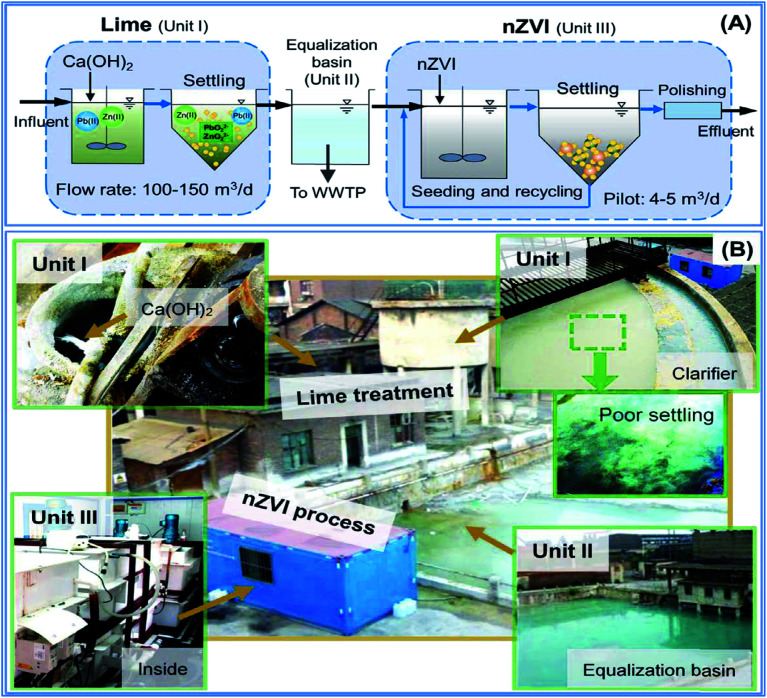
Pilot scale test lead using nZVI & lime; adapted from Wang *et al.* (2016).^68^

Another field study was conducted by Wang *et al.* (2016)^44^ to evaluate a multi-stage system consisting of lime (eqn (16)) and nZVI processes for the removal of Pb(ii) and Zn(ii) in wastewater generated by a smelting plant in China. The wastewater contained highly hazardous metal ions (*e.g.*, Pb(ii)). The treatment system was constructed on-site, and the study was conducted for a period of 6 days. Wang *et al.* (2016) designed pilot test reactors to compare the removal of lead and zinc using nZVI line separately (Fig. 11).

The nanoscale zero-valent iron (nZVI) was compared with lime (Ca(OH)_2_), the most widely used heavy metal precipitant, for Pb(ii) and Zn(ii) removal field experiments (Fig. 11). The test result was verified in a field continuous-flow experiment using tons of lime and kilos of nZVI. The results show that sub-ppm levels (∼0.1 mg L^−1^) of Pb(ii) could not be achieved *via* lime precipitation alone (eqn (16) and (17)) due to the solubility limits of lead hydroxide and its re-dissolution (eqn (18)) caused by the strong alkalinity of lime.^44^ In comparison, stable and lower levels of Pb(ii) were easily attained using nZVI due to its multifunctional properties and good tolerance for influent fluctuation enabled by its inherent pH-stabilizing nature (Table 3).

**Table 3:** Comparative analysis of the removal of heavy metals using lime and nZVI (Wang *et al.*, 2016)

Parameters	Influent	After lime treatment	After nZVI treatment	Final effluent
pH	2.5	5–13	8.5	7.3
Pb(ii) conc. (mg L^−1^)	610	19.1	0.05	<0.08
Cu(ii) + Cd(ii) conc. (mg L^−1^)	265	2.0	0.04	<0.02

According to the study conducted by Wang *et al.* (2016), the concentrations of the heavy metals (lead and copper) were much lower than those predicted by the theoretical solubility of metal hydroxides; hydroxide precipitation cannot solely account for the efficient removal, and the combined effect of sorption (eqn (20)), (co)precipitation (eqn (17)) and chemical reduction (eqn (19)) in the system leads to highly efficient metal removal that cannot be achieved *via* hydroxide precipitation.

16 Ca(OH)_2(s)_ → Ca^2+^ + 2OH^+^

17 (Co)precipitation: Pb^2+^ + 2OH^−^ → Pb(OH)_2_↓

18 Re-dissolution: Pb(OH)_2(s)_ + 2OH^+^ → Pb(OH)_4_^2−^

19 Reduction: Fe^0^ + Pb^2+^ → Fe^2+^ + Pb^0^

20 Sorption: ≡Fe–OOH + Pb^2+^ → ≡Fe–OOPb + 2H^+^

### 6.3 Chemical reduction methods for copper removal from copper industry

**Fig. 12:**
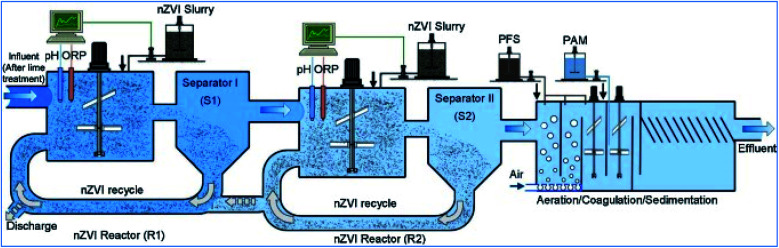
Pilot nZVI technology based heavy metal removal in Jiangxi, China operated by the Jiangxi Copper Company ltd; adapted from Li *et al.* (2017b).^69^

Li *et al.* conducted a pilot test to evaluate nZVI efficiency for various heavy metal removal at the nZVI technology-based treatment facility, located in Jiangxi, China and operated by the Jiangxi Copper Company, which showed a promising result for copper ion removal from copper containing wastewater.^69^ The nZVI treatment plant was constructed in April, 2012, and served as a pre-treatment to remove arsenic and other metals such as copper(ii) from the wastewater (Fig. 12). The treated wastewater was then pumped to a centralized wastewater treatment facility and mixed with other waste streams for further removal of organic contaminants. The nZVI reactors were constructed as separate modules. Each nZVI reactor has dimensions of 4.8 m (height) × 4.8 m (length) × 3 m (width), and has an effective volume of approximately 60 m^3^. pH and Eh in the reactors are monitored continuously using online electrodes of pH and ORP. The ORP values are corrected with respect to the Ag/AgCl reference electrode and converted to the standard electrode potential (Eh). Wastewater samples were collected daily at the outlet of each treatment unit in the process, such as the outlets of equalization basin and nZVI reactor. Solid samples (spent nZVI) were collected from the nZVI reactor. According to the pilot test, quick removal of the four target ions was observed after nZVI addition; 99% of copper, 90% of arsenic, zinc and nickel were removed in less than 10 min. The removal efficiencies are all greater than 96% after 60 min. Increasing nZVI dosages improves the effluent quality and the concentrations of the target ions after filtration meet the local discharge guidelines. The fast and simultaneous removal of different heavy metals using nZVI is the major reason that nZVI was selected and approved for field application.

## 7. Limitations and prospects of nZVI application

Studies showed that nZVI can be adsorbed on cell membranes of bacteria, or penetrate through them, which often leads to disturbances in the functioning of the cell.^70^ Nanoparticles adsorbed on cell membranes can block cellular ducts, cause structural changes to the membranes, or inhibit mobility and nutrient intake and result in death of the bacteria.

Another often-suggested mechanism contributing to nZVI toxicity involves the generation of highly reactive oxygen species, which accumulate in the cell environment and denature macromolecules including lipids, proteins, and nucleic acids, damaging intracellular structures and eventually leading to cell death. Because under aerated conditions, Fe oxidizes more rapidly than it would under anaerobic conditions, the contribution of Fe^2+^ to nZVI toxicity is higher under anaerobic conditions than under aerobic conditions.^71^ Research suggested both modifications and limiting the direct contact of the nZVI surface with cells to reduce the toxicity.

The application of nZVI works best under anaerobic conditions since it is otherwise quickly transformed to iron oxides under aerobic conditions. Any modification to the surface such as oxidation would therefore affect the performance of the particles.^14^ Some of the other major challenges identified with the use of nZVI are the rapid aggregation of the particles, passivation (quick oxidation by non-target compounds), sorption to other materials and rapid sedimentation (because of its high density/7800 kg m^−3^) that consequently limits the mobility of nanoparticles in the aquatic environment.^15,25^

Pure nZVI agglomerate easily. It is also very sensitive towards oxidation and reduces the electron donation during remediation of heavy metal containing wastewater. nZVI can be prepared in many ways, however, it is easily oxidized and difficult to preserve. Many of the preparation methods take place under strict conditions, and hence, it is very difficult to proceed to large scale production. However, in general, it is a very promising reductant for cationic pollutants such as metal ions and cationic dyes, which can also be used in other heavy metal remediation. The heavy metal removal mechanisms are limited not only to reduction and adsorption but also to oxidation. The Fenton reaction taking place in the aqueous solution change Fe^3+^ to Fe^2+^ and gives an opportunity for the targetted pollutants to be oxidized.^72^

## 8. Conclusion

For the treatment of contaminated water, growing theoretical and empirical evidence has proven nZVI as both highly effective and versatile. The sorption, complexation and reduction processes played a great role for the removal of heavy metals. In recent years, there have been significant innovations in terms of manufacture techniques, physicochemical functionalisation and enhancements in sub-surface stability of nZVI. The tests that have been done on the removal activity of Pb, Cd and Cu showed the possibility of applying ZVI nanoparticles at field scale. However, some of its properties such as susceptibility to oxidation and formation of iron oxide, aggregation and sediment-ability need to be studied.

Various pilot scale tests for the removal of heavy metals have been conducted. All the tests followed the concept of green chemistry to treat wastewater and recover heavy metals. Practically, the pilot test was operated in continuous mode. To make the process affordable, nZVI was recirculated and reused. The nZVI process presented is designed for large-scale applications and is easy-to-operate. The results of the reviewed practical pilot test suggested that this nZVI process is much more efficient than conventional technologies of wastewater treatment, and some economic minerals such as copper could be easily recovered from the wastewater using this process. In general, the technology has showed satisfactory and promising results for large scale industrial wastewater treatment.

## Author contributions

Mekonnen Maschal Tarekegn has contributed in compiling articles/literatures, reviewing and manuscript preparation.

Andualem Mekonnen Hiruy and Ahmed Hussen Dekebo have contributed in reviewing and correcting the manuscript.

## Conflicts of interest

We declare that there are no conflicts related to this article.

## Supplementary Material
