# Correction: Nano zero valent iron (nZVI) particles for the removal of heavy metals (Cd^2+^, Cu^2+^ and Pb^2+^) from aqueous solutions

**DOI:** 10.1039/d1ra90135d

**Published:** 2021-08-09

**Authors:** Mekonnen Maschal Tarekegn, Andualem Mekonnen Hiruy, Ahmed Hussen Dekebo

**Affiliations:** Ethiopian Civil Service University, Department of Environment and Climate Change Addis Ababa Ethiopia Mekonnen.maschal@ecsu.edu.et maschalm12@gmail.com; Addis Ababa University, College of Natural and Computational Sciences, Centre for Environmental Science Addis Ababa Ethiopia

## Abstract

Correction for ‘Nano zero valent iron (nZVI) particles for the removal of heavy metals (Cd^2+^, Cu^2+^ and Pb^2+^) from aqueous solutions’ by Mekonnen Maschal Tarekegn *et al.*, *RSC Adv.*, 2021, **11**, 18539–18551. DOI: 10.1039/D1RA01427G.

The authors regret that reference 48 was incorrect. The correct reference is given below as reference 1.^1^

The Royal Society of Chemistry apologises for these errors and any consequent inconvenience to authors and readers.

## Supplementary Material
